# Content validity and test-retest reliability of a low back pain questionnaire in Zimbabwean adolescents

**DOI:** 10.1186/s40945-017-0031-y

**Published:** 2017-02-28

**Authors:** Matthew Chiwaridzo, Tafadzwa Nicole Chikasha, Nirmala Naidoo, Jermaine Matewu Dambi, Cathrine Tadyanemhandu, Nyaradzai Munambah, Precious Trish Chizanga

**Affiliations:** 1 Rehabilitation Department, University of Zimbabwe, College of Health Sciences, P.O Box A178, Avondale, Harare Zimbabwe; 2 Division of Physiotherapy, University of Cape Town, School of Health and Rehabilitation Sciences, Faculty of Health Sciences, Cape Town, South Africa; 3 Department of Tourism and Hospitality, Harare Polytechnic College, P.O Box CY 407, Causeway, Harare Zimbabwe

**Keywords:** Adolescents, Content Validity, Low Back Pain, Test-retest reliability, Zimbabwe

## Abstract

**Background:**

In Zimbabwe, a recent increase in the volume of research on recurrent non-specific low back pain (NSLBP) has revealed that adolescents are commonly affected. This is alarming to health professionals and parents and calls for serious primary preventative strategies to be developed and implemented forthwith. Early identification initiatives should be prioritised in order to curtail the condition and its progression. In an attempt to be proactive in minimising the prevalence of recurrent NSLBP, this study was conducted to evaluate the content validity and test-retest reliability of a survey questionnaire with the aim of proffering a valid and reliable questionnaire which can be used in non-clinical settings to identify adolescents with recurrent NSLBP in Harare, Zimbabwe and determine the possible factors associated with the condition.

**Methods:**

The study was conducted in two parts. The first part assessed content validity of the questionnaire using four experts derived from academia and clinical practice. The second part evaluated the reliability of the questionnaire among 125 high school-children aged between 13 and 19 years in a test-retest study.

**Results:**

Twenty-six (26) out of thirty questions in the questionnaire had an Item Content Validity index of 1.00, demonstrating complete agreement among content experts. Overall, the Scale Content Validity Index for the questionnaire was 0.97. Item completion for the reliability study was satisfactory. The questionnaire items had kappa values ranging from 0.17 (slight agreement) to 1 (perfect agreement). High levels of reliability were found for the questions on school bag use (*k*=0.94), sports participation (*k*=0.97), and lifetime prevalence (*k*=0.89).

**Conclusion:**

Excellent content validity and slight to perfect test-retest reliability was found for the Low Back Pain (LBP) questionnaire. These results are comparable to findings of other studies evaluating the psychometric properties of LBP questionnaires. Cognisant of the limitations of the study, the results of this study suggest that the LBP questionnaire could be used in local studies investigating LBP among adolescents although questions enquiring on functional limitations and sciatica may need further consideration.

**Electronic supplementary material:**

The online version of this article (doi:10.1186/s40945-017-0031-y) contains supplementary material, which is available to authorized users.

## Background

Low back pain (LBP) is a highly prevalent health problem worldwide [1]. In the last decade, there has been an increase in the volume of research highlighting the problem of LBP in adolescents [2–5]. It has become established that LBP is a common occurrence in adolescents just as in adults with the majority of the episodes being non-specific in nature [6, 7]. Although self-limiting in many instances, a small subset of adolescents experience significant recurrent episodes [6–8]. Epidemiological studies estimate the prevalence of recurrent non-specific low back pain (NSLBP) in adolescents to be between 8% and 36% and link the condition to biological, mechanical, psychological, and lifestyle-related factors [8–17].

The consequences of recurrent NSLBP in adolescents are well documented in the literature. Unabated, the condition negatively affect the health-related quality of life (HRQoL) leading to school absenteeism and functional limitations [18–21]. In a bid to seek symptomatic relief from the chronic symptoms, adolescents may consult health-care professionals. A study conducted by Masiero et al. [18] among 7 542 Italian adolescents between the ages of 13 and 15 years showed that LBP sufferers regularly consulted health-care professional for their symptoms.

Additionally, adolescent recurrent NSLBP has been linked to disc degenerative changes in the spine [8, 19]. In a cross-sectional cohort study of 439 children aged 13 years, results from magnetic resonance imaging (MRI) showed that reduced signal intensity and irregular nucleus shape in the upper lumber discs were significantly associated with reports of LBP [19]. Although the clinical relevance of these abnormal findings may not be clear, spinal degenerative changes in young population of adolescents should be a cause of concern.

With longitudinal epidemiological evidence linking adolescent recurrent NSLBP to chronic debilitating LBP in adulthood, this further raise a lot of questions on the most effective prevention strategies to curtail the transitioning [8, 12, 21]. In addition, this evidence creates a huge need to be proactive in primary prevention strategies during the preadolescent and/or adolescent period in a bid to minimise the transitioning into a chronic, disabling and costly condition in adults. From a public health perspective, this call for proactivity is particularly important for low-income countries such as Zimbabwe with constrained health-care resources and LBP health matters may not be prioritised relative to other conditions such as HIV/AIDS.

In Zimbabwe, the problem of LBP among adolescents is a recent or emerging epidemiological phenomenon. This evidenced by a sudden increase in the number of published studies investigating recurrent NSLBP in adolescents since 2014. Evidence from cross-sectional studies conducted so far have highlighted the existence, magnitude and the factors associated with recurrent NSLBP among Zimbabwean adolescents in high schools [11, 17, 22–24]. The 12-month prevalence figures reported for recurrent NSLBP are relatively high ranging between 28.8 to 30.7% [11, 24]. The existence of this problem among supposedly ‘healthy’ adolescents should be alarming to health professionals, teachers and parents. The fact that high school adolescents are affected before they begin their work life is disturbing considering the recurrent nature of the condition and the critical developmental changes occurring in the spines of adolescents [25]. As a first step in preventative efforts, it seems logical and proactive in validating a survey LBP questionnaire designed to screen for symptoms of recurrent NSLBP and determine the factors associated with the condition in our context. To our knowledge, no study has been conducted with a special focus on validating a survey instrument designed for the investigation of LBP problems among the young population in Zimbabwe. Paucity of a reliable and validated questionnaire in a country with a reported high prevalence rate of adolescent recurrent NSLBP pain is a significant shortcoming to the need to curtail the problem [11]. Such instruments could help researchers, health and non-health professionals in identifying high school adolescents with a greater need for early intervention in non-clinical settings.

This study was conducted, therefore, to document the evidence of content validity and reproducibility of a LBP survey questionnaire developed to identify adolescents with recurrent NSLBP and identify the factors associated with the condition. The specific objectives of this study were to determine the Item Content Validity Index (I-CVI) of each question in the questionnaire and henceforth calculate the average Scale Content Validity Index (S-CVI/Ave) for the questionnaire. Secondarily, the study sought to determine the kappa coefficient (k) for each ordinal item in the questionnaire and the Intraclass Correlation Coefficient (ICCs) for each question eliciting continuous responses as a measure of test-retest reliability.

## Methods

### Study design, research setting and participants

Broadly, this study was conducted in two parts. The first part was conducted to establish the content validity of the developed questionnaire from a cross-sectional sample of experienced experts. As reported in the literature, content validity assesses whether an instrument adequately or exhaustively contains all the items necessary to represent the concept being measured [26]. Subsequently, the second part of the study was conducted to evaluate the reliability of the content-validated LBP questionnaire using a test-retest study design.

Participants for the content validation study were experts recruited from various medical departments at the University of Zimbabwe, College of Health Sciences (UZCHS) and government-owned tertiary health-care institutions in Harare, Zimbabwe. The UZCHS is the only institution that offers training to all health care professionals in the country. There are three state tertiary health care institutions serving as referral centres in Harare namely, Parirenyatwa Group of Hospital, Harare Central Hospital and Chitungwiza Central Hospital.

Participants for the reliability study were high school learners recruited from government-administered high schools in Harare, Zimbabwe. Of the 55 government high schools in Harare, which are divided into S_1_ category (17 schools) and S_2_ (38 schools), only three schools (One from S_1_ and Two from S_2_) were randomly selected, based on a strategy that considered ratio of schools in each category. At the time of data collection, there were 3 246 registered full-time school-children enrolled at these three selected high schools. However, with the high school education system in Zimbabwe made up of two levels, Ordinary level education (Form 1 to 4) and Advanced level education (Form 5 & 6), two independent forms were randomly selected from each of the three selected schools. This gave a total of six different classes with a total of 180 students all eligible for participation in the study.

### Eligibility criteria for the reliability study

Participation was based on students’ expression of willingness to participate evidenced by signing of the assent form. In addition, only students with parental/legal guardian permissions through signing of the informed consent form were recruited. To further select appropriate students, the exclusion criteria previously used in other related cross-sectional studies investigating LBP problems among adolescents in the local setting was adopted. Students with parental reports on the Adolescent Medical Health Questionnaire of spinal, neurological, orthopaedic conditions and any previous report of trauma to the back, hip and knees were excluded [11, 22]. Participants were also excluded if they had overt (based on the researcher perceived judgement) or covert (according to them or their parents’ reports) physical deformities such as leg length discrepancy, scoliosis and lordosis as they could be associated with specific LBP [11, 22–24].

### Low back pain questionnaire

The LBP Questionnaire was developed in English language mainly to determine the prevalence of recurrent NSLBP and investigate the factors associated with the condition among a cross-sectional sample of high school Zimbabwean adolescents. The choice of a questionnaire as a survey instrument was based on the reported fact that pain is a subjective experience and 85-90% of school-children can reliably report their pain experience [5, 27, 28]. Although detailed information on the development of the questionnaire falls outside the scope of this paper, briefly the questionnaire was developed through obtaining questions from previously validated instruments from literature [9, 25, 28–31]. The process of developing a questionnaire was guided by a checklist adopted from Boynton and Greenhalgh [32]. Additionally, an attempt was made to identify questions that fitted into pre-selected domains of a conceptual model propounded by Wilson and Cleary such as symptom status, biological and physiological variables, functional status, and characteristics of the individual [33].

The questionnaire developed contained four sections with a total of 30 questions (see Additional file 1). Briefly, section A gathered information primarily on the prevalence of recurrent NSLBP and its definitional parameters with regards to frequency, duration, intensity of episodes and the presence or absence of sciatica (radiating leg pain). The Visual Analogue Scale (VAS) was used to quantify pain intensity. A Delphi agreed definition of recurrent NSLBP adopted from Stanton et al. [34] was used to identify participants with recurrent cases in the last 12 months. In addition, this section had questions on lifetime prevalence (LBP at some point in life), point prevalence (LBP at the time of completing the questionnaire) and the perceived consequences of recurrent NSLBP (functional limitations and school absenteeism). Nine items adapted from Hanover Functional Ability questionnaire were used to enquire about functional limitations related to recurrent NSLBP in adolescents. This instrument is widely used in literature as a measure of disability among adolescents with LBP [13, 17, 35, 36]. The health-seeking behaviour for the adolescents with recurrent NSLBP was also ascertained. This behaviour described seeking either formal or informal health services for the recurrent symptoms of NSLBP [17].

The subsequent sections, B, C and D contained questions on factors associated with the recurrent NSLBP. Section B elicited information on school-bag related factors (school-bag use, duration of carriage, method of carriage and perceived perception of school-bag weight). Section C had questions on physical activity (sports participation, frequency, sport played and sedentary lifestyle). Section D asked about lifetime and recent (in the past week) smoking status of the adolescents. These pre-selected factors were included in the questionnaire on the basis that they had been reported in previous studies as significant factors associated with LBP among adolescents [16, 37–42].

### Procedure

#### Ethical and institutional approvals

After obtaining institutional approvals from the Ministry of Primary and Secondary Education (C/426/3) and Harare Provincial Educational Office (G/42/1), ethical approval was sought and obtained from the Joint Research Ethics Committee for the University of Zimbabwe College of Health Sciences and Parirenyatwa Group of Hospitals (JREC/254/15) and the Medical Research Council of Zimbabwe (MRCZ/B/961). Further approvals to access the schools were obtained from the respective headmasters of the three participating schools. Content experts had to submit a signed informed consent form indicating willingness to participate.

#### Pre-testing of the instrument

Pre-testing of the English LBP Questionnaire was conducted at one of the selected schools after obtaining ethical and institutional approvals. Twenty (20) randomly selected Form Three (3) students (mean age=15.5±2.6 years) volunteered to participate in the preliminary study. The students were deliberately omitted in the main study data collection. The primary objectives of the pre-test study were to assess the comprehensibility of the questionnaire and evaluate the feasibility concerns for the main study data collection. The maximum amount of time needed to complete the questionnaires by all the participants was used as a measure of comprehensibility.

The procedure for the pre-test involved giving information letters explaining the purpose and the nature of the pre-test study to the respondents first. The researcher (TNC) augmented the information by clear oral explanations. Thereafter, the questionnaires were self-administered in the presence of the class teacher. While completing the questionnaires, respondents were strongly encouraged to ask the researcher questions when clarification was necessary. At the end, respondents were requested to comment whether they understood each question in the questionnaire and the response options provided. Any question, response option, word or phrase that was misunderstood by at least one of the respondents was modified or reworded by the researcher until it was deemed satisfactory by the respective respondent(s).

On average, the respondents took 40±6 minutes to complete the questionnaire which was more than the anticipated 10±5 minutes. This was attributable to a number of reasons. The participants seemed elated to be participating in the study resulting in unceasing deliberations and stifled laughs amongst themselves whilst answering the questionnaire. This was particularly noticed on questions enquiring about LBP status, smoking status and sports participation. This was despite several attempts by the researcher and the class teacher encouraging independency in completing the questionnaires. Another significant reason noted for the lengthened time of completing the questionnaires was the frequent interruptions made by the respondents seeking clarification from the researcher. This happened on a number of questions such as the question on pain intensity for recurrent NSLBP, sciatica, and functional limitations, type of sports or exercise and smoking status.

Overall, there were no changes suggested by the respondents on the nature of the questions in the questionnaire and there were also no changes in the total number of question items following the pre-testing. However, there were lessons drawn from the pre-test study which informed the design of the main study data collection procedure worth mentioning:

1. The research team had to ensure that respondents were organised in sitting arrangements that does not allow for copying or deliberate discussions of their responses.
2. It was important for the research team to have a class teacher present during data collection to ensure that respondents conduct themselves cordially and professionally;
3. It was important for the research team to communicate with the school authorities prior to their data collection visit either by telephone or a physical visit to the school to arrange for time and place for data collection, class of students eligible for the main study and possibly negotiate for local assistance from the class teachers for the data collection;
4. It was important to translate the English questionnaire into a local language and for the main study data collection to allow the respondents to choose a questionnaire in a language they prefer for ease of understanding.

#### Content validation of the instrument

Content validation of the instrument was conducted after the pre-test study between December 2015 and March 2016. Although literature is controversial on the ideal number of content experts needed in a validation study, invitational letters were sent to seven independent content experts soliciting their participation. The content experts were nominated on the basis of either experience in epidemiological and/or musculoskeletal research as evidenced by the number of publications in that field or significant clinical work experience in LBP management whether in medicine, orthopaedic surgery, neurology and rehabilitation from the three large health-care facilities.

The content experts were given one week to respond to our request to participate in the study. Those that indicated willingness to participate were thereafter furnished through email with an informed consent form attached to an information cover letter, the English version of survey questionnaire, a brief demographic questionnaire and the evaluation criteria form. The information cover letter explained the purpose of the study, the reasons for the selecting the content expert, a description of the questionnaire and an explanation on the content evaluation procedure. Each expert was asked to assess the relevance of each question in the instrument. This meant that experts had to assess whether all the items in the LBP Questionnaire refer to relevant aspects of the construct of recurrent NSLBP being measured. In addition, experts had to assess whether all the items were relevant for the target population and the intended purpose of the questionnaire. To judge relevance of each question, a 4-point scale based on a criteria propounded by Davis was used [43]. The experts scored each question as follows: *1 = not relevant, 2 = somewhat relevant, 3 = quite relevant, 4 = highly relevant.*

The experts were specifically requested to provide recommendations (for revision or deletion) for each question which they would have scored low (1 or 2). For the questions in need of revision, the experts had to comment on the clarity (how clearly the question was worded) and were requested to provide a possible option of ensuring the relevance of the question. The experts were given a maximum period of two weeks to validate the questionnaire and return through email or in person to the researcher (TNC). Reminders would be sent through emails and short messaging service (SMS) after every three days to maximize the response rate. At the end of the validation, responses from the experts were analysed by one of the researchers (MC).

#### Translation of the instrument

After the LBP Questionnaire was content-validated and necessary changes effected as suggested by the content experts, the questionnaire was translated into Shona, a local language spoken in Harare, Zimbabwe. The procedure for the translation process was informally guided by the suggestions from a study by Sousa et al. [44]. Two qualified independent translators (PTC and SC) were used for the forward translation striving for word-for-word translation. PTC had considerable background knowledge on medical terminology and SC neither had medical knowledge of the construct and the health-care terminology. Using a committee approach composed of three independent reviewers (LM, SM and SS) and one of the researchers (MC), the two versions were assessed and compared for conceptual equivalences, differences and discrepancies in the sentence structures of instruction, questions and response options. Major discrepancies were shared and discussed among the reviewers until a consensus was reached. For the back translation of the Shona questionnaire to English, two other independent expert translators (TM and CJ) were employed. The translators were blinded to the specific purpose of the back translation which was to compare the back-translated English questionnaire with the originally developed English questionnaire. The first translator (TM) was chosen on the basis of smattering knowledge about medical terminology and content area. The second translator was employed purely for her cross-cultural translational and linguistic abilities. The two versions engendered were assessed and compared by a committee composed of two research team members (TNC and MC) who were primarily involved in the initial design of the questionnaire and one independent author (CT). Discrepancies were discussed and resolved through consensus among the committee members to derive a final version of the instrument.

#### Test-retest reliability

The test-retest reliability study was conducted after the content validation and translation of the English questionnaire into Shona. Initially, parental documents (information letters, informed consent forms, Adolescent Medical Health Questionnaires) were sent to parents/guardians of eligible children. The school-children were given seven days to return the documents to their school teacher with the written informed consent forms signed and the Adolescent Medical Health Questionnaire completed. The Adolescent Medical Health Questionnaire was used to establish the medical history of the participating children as reported by parents. The questionnaire was adopted from previous studies and it provided the basis for excluding school-children not fulfilling the inclusion criteria [11, 17, 23, 24].

After obtaining parental permissions, the researcher (TNC) visited the three participating schools consecutively for data collection. The questionnaires were self-administered in classrooms to eligible students in the presence of the class teacher. Standardised oral instructions were given to participants to supplement the information regarding the study provided on information letters. Participants had the option of either choosing the English or the translated Shona questionnaire. Participants were free to ask questions for clarification purposes when necessary. The students sat approximately 50cm apart from each other and were strictly instructed not to discuss their responses with one another. During the initial test, the participants were not told that they will be re-tested after seven days.

After the seven days, the questionnaires were self-administered again to the participating school-children, present at school and in class, using the same procedures described for the test study and completing the same language version of the questionnaire as previous. The time interval of seven days was chosen to reduce the possibility of participants remembering their initial responses, the so-called “carry-over” effect and to lessen the possibility of the LBP changing between the tests [45]. It is a well-known fact that LBP is characterised by unpredictable patterns of recurrences and remissions with a recurrent episode lasting approximately seven days [7, 11].

#### Statistical analysis

Content experts had to rate the relevance of each question/item in the questionnaire on a scale of 1 to 4. One proportion agreement method, the Content Validity Index (CVI), was used to estimate quantitatively the content validity [46, 47]. Specifically, the Item Content Validity Index (I-CVI) was computed as the number of content valid experts giving a rating of either 3 (quite relevant) or 4 (highly relevant), divided by the total number of experts [48, 49]. Because the I-CVI should be 1.00 when four or fewer experts are used to judge the validity of each question [47], total agreement (the number of items that achieved the I-CVI of 1.00 divided by the total number of items to be validated in the questionnaire) was calculated to represent the proportion of questions that experts deemed highly or extremely relevant. Additionally, a Scale-Content Validity Index (S-CVI/Ave) was computed to summarise the overall content validity of the questionnaire. This was calculated as the average I-CVIs for all the items in the questionnaire. An S-CVI/Ave of greater than 0.90 would qualify the questionnaire to be content valid [43].

For the test-retest reliability study, descriptive statistics were used to describe baseline demographic characteristics of the respondents. Statistical analyses were performed using STATISTICA version 13. Data normality for continuous variables such as age was assessed using the Kolmogorov Smirnov and Lilliefors test. The *t*-test independent by groups was used to compare the mean age of onset for LBP as well as the mean intensity of recurrent NSLBP by gender. The significance level was fixed at *p*<0.05. Item completion was evaluated and percentage agreement calculated for each question as the number of agreement scores divided by the total number of scores between studies [50]. Accounting for agreement occurring by chance, test-retest reliability was further evaluated using Cohen’s kappa coefficient (k) [51]. Cohen’s weighted kappa (Kw) was used for items with more than two possible responses [45]. The kappa coefficients were interpreted using the criteria outlined by Landis and Koch [52] summarised as follows: <0 (poor agreement); 0-0.2 (slight agreement); 0.21-0.40 (fair agreement); 0.41-0.60 (moderate agreement); 0.61-0.80 (substantial agreement); 0.81-1.0 (almost perfect agreement). In addition, the standard error of kappa (SE_k_) and the corresponding 95% Confidence Interval (CI) for each obtained kappa value were also computed. For continuous data (Q2, 6 and 9), reliability was analysed using Intraclass correlation coefficient (ICC) and values were reported with the corresponding 95% CI. The ICCs expressed the absolute agreement between single measures on a two way mixed model (3, 1). ICCs values above 0.7 were considered high [53]. In addition, the standard error of measurement (SEM) was also computeds as SD x (square root of (1-ICC). SPPS Version 23 was used for this analysis. The dependent sample *t*-test was used to compare the mean age of onset of LBP, the mean pain intensity for recurrent NSLBP and the mean pain intensity for point prevalence pain between the two assessments.

## Results

### Content validation

Out of the seven content experts invited to participate in the study, four agreed to participate. Three were in academia and were lecturers from the University of Zimbabwe, College of Health Sciences from the Department of Rehabilitation, Nursing Science and Community Medicine. The other expert was a senior musculoskeletal physiotherapist at Harare Central Hospital, who at the time of the study was enrolled as a third year PhD candidate. The mean age of the experts was 42.8 (SD=8.14) years. The number of questions in the LBP survey questionnaire remained unchanged after the validation process. I-CVIs of each item in the questionnaire are shown on Table 1. Briefly, I-CVI for the survey questionnaire ranged from 0.75 to 1.00. Twenty-six (26) out of thirty questions in the questionnaire had an I-CVI of 1.00, demonstrating complete agreement among the content experts. The calculated S-CVI/Ave for the questionnaire was therefore 0.97.

**Table 1:** Results for content validation of the low back pain survey questionnaire by content experts

Item number	Question description	Expert raters				I-CVI^a^
		1	2	3	4	
Q1	Lifetime prevalence	4	4	4	4	1.00
Q2	Age of onset	4	4	4	4	1.00
Q3	Recurrent NSLBP^c^	4	4	4	2	0.75
Q4	Frequency of recurrent NSLBP	4	4	4	3	1.00
Q5	Duration of recurrent NSLBP	2	4	4	4	0.75
Q6	Intensity of recurrent NSLBP	4	4	4	4	1.00
Q7	Sciatica	4	4	4	4	1.00
Q8	Point prevalence	4	4	4	4	1.00
Q9	Point pain intensity	4	4	4	4	1.00
Q10	Medical treatment	4	4	4	4	1.00
Q11a	Sitting on a chair	4	4	4	4	1.00
b)	Reaching up	4	4	4	4	1.00
c)	Standing	4	4	4	4	1.00
d)	Walking	4	4	4	4	1.00
e)	Sporting activity	4	4	4	4	1.00
f)	Bending	4	4	4	4	1.00
g)	School-bag carrying	4	4	4	4	1.00
h)	Sitting up in bed	4	4	4	4	1.00
i)	Running	4	4	4	4	1.00
Q12	Absenteeism	3	4	4	4	1.00
Q13	Bag carriage	4	4	4	4	1.00
Q14	Perception school-bag weight	4	4	4	3	1.00
Q15	Duration of carrying school-bag	3	4	4	4	1.00
Q16	Method of carrying school-bag	4	4	4	4	1.00
Q17	Sports participation	4	4	4	4	1.00
Q18	Type of sport	4	4	4	4	1.00
Q19	Sports duration	4	4	4	4	1.00
Q20	Sedentary time	3	4	4	4	1.00
Q21	Smoking	4	4	4	1	0.75
Q22	Past week smoking	4	4	4	1	0.75
	^d^Total agreement=0.86 ^b^S-CVI/Ave = 0.97					

^a^I-CVI (Item content validity index) =computed as the number of content valid experts giving a rating of either 3 (quite relevant) or 4 (highly relevant), divided by the total number of experts.

^b^S-CVI/Ave (scale content validity index) =computed as the average I-CVIs for all the items in the questionnaire.

^c^NSLBP= non-specific low back pain

^d^Total agreement= the number of items that achieved the I-CVI of 1.00 divided by the total number of items to be validated in the questionnaire

### Test-retest reliability study

#### Participation rate

**Fig. 1:**
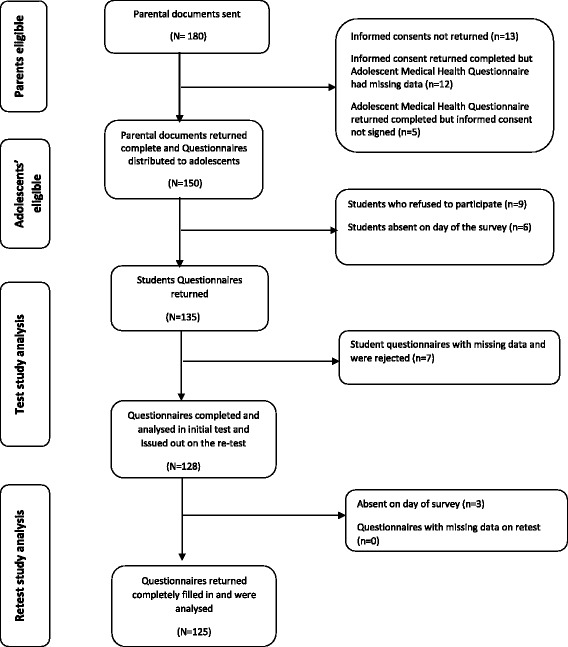
Flow chart showing the distribution of the questionnaires in the test-retest reliability study

Out of 180 eligible participants, 150 had the permission to participate from their parents and gave their consent. However, nine (9) of the eligible students refused to participate volitionally. In total, sixteen (16) students were excluded because they were absent at the first or second day of the survey or because they did not complete the questionnaire leaving a lot of questions unanswered. One-hundred and twenty-five (125) questionnaires were completely filled at both the test and retest assessment and were used for all analyses (Fig. 1).

#### Sample characteristics

**Fig. 2:**
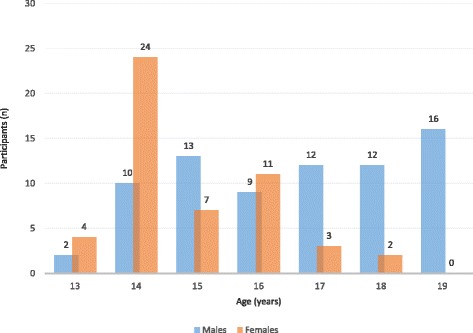
The number of participants in the test-retest reliability study by gender and age (*N*=125)

Age data of participants was normally distributed (KSd= 0.17, *P*< 0.01; Lilliefors *P*< 0.01). The mean age of the sample participants was 15.9 (SD=1.9) years and age range was 13 to 19 years. The sample had 74 (59.2%) male participants who were significantly older compared to females [t (123) =5.7, *p*< 0.001]. Figure 2 shows the number of participants according to age and gender.

#### Reliability results

Item completion for the questionnaire was satisfactory for the test-retest reliability study. There were very few missing data and the results are reported with missing data questionnaires excluded. As expected, demographic characteristics (age, gender, place of residence, and level of education) were consistent between the two tests. Table 2 details the test-retest reliability results for the low back questionnaire. Briefly, the LBP questionnaire had *k* values ranging from 0.17 (slight agreement) to 1 (perfect agreement). Almost perfect to perfect agreements were found for the items that evaluated school bag use (*k*=1), sports participation (*k*=0.97), lifetime prevalence (*k*=0.89), smoking status (*k*=0.84) and perceptions of school bag weight (*k*=0.81). The screening question for recurrent NSLBP showed substantial agreement (*k*=0.78) so as questions related to the frequency and duration of recurrent NSLBP episodes. The questionnaire items adopted from Hanover Functional Ability Questionnaire evaluating functional consequences had low kappa values ranging from 0.17 (slight) to 0.43 (moderate). However, the question eliciting information on the health-seeking behaviour of school-children with recurrent NSLBP had substantial agreement (*k*=0.73). The question on school absenteeism achieved moderate reliability (*k*=0.45). Intraclass correlation coefficients (ICCs) for questions eliciting continuous responses showed variable reliability values for the questions on age of onset for LBP (ICC=0.87), pain intensity for recurrent non-specific (ICC=0.97) and point prevalence pain (ICC=0.62) (Table 3).

**Table 2:** Test-retest reliability results for the Low Back Pain Questionnaire (*N*=125)

Item number	Item description	Percentage agreement (%)	Kappa coefficient (SE_k_)	95% CI
Q1.	Lifetime prevalence	96.0	0.89 (0.04)	0.81-0.99
Q3.	Recurrent NSLBP	87.8	0.78 (0.06)	0.66-0.90
Q4.	Frequency of recurrent NSLBP	82.9	0.71 (0.11)	0.49-0.93
Q5.	Duration of recurrent NSLBP	97.0	0.65 (0.32)	0.02-1.00
Q7.	Sciatica	58.5	0.20 (0.12)	0.00-0.44
Q8.	Point prevalence	88.7	0.38 (0.13)	0.13-0.63
Q10.	Medical treatment	95.7	0.73 (0.18)	0.37-1.00
Q11a)	Sitting	70.2	0.38 (0.13)	0.12-0.64
b)	Reaching up	78.7	0.37 (0.13)	0.10-0.63
c)	Standing	61.7	0.22 (0.14)	0.00-0.50
d)	Walking	68.1	0.37 (0.12)	0.12-0.63
e)	Sporting activity	68.1	0.36 (0.14)	0.10-0.63
f)	Bending	72.3	0.43 (0.14)	0.16-0.69
g)	School-bag carrying	72.3	0.39 (0.14)	0.11-0.67
h)	Sitting	72.3	0.33 (0.16)	0.03-0.64
i)	Running	70.2	0.17 (0.16)	0.00-0.50
Q12.	Absenteeism	91.5	0.45 (0.23)	0.00-0.97
Q13.	Bag Carriage	99.2	0. 94 (0.03)	0.88-1.00
Q14.	Perception of school bag weight	91.2	0.81 (0.06)	0.70-0.92
Q15.	Duration of carrying school bag	75.0	0.76 (0.04)	0.68-0.84
Q16.	Method of carrying school bag	91.9	0.61 (0.11)	0.40-0.83
Q17.	Sports participation	98.1	0.97 (0.03)	0.91-1.00
Q19.	Sports duration	79.2	0.69 (0.05)	0.69-0.88
Q20	Sedentary time	73.6	0.77 (0.04)	0.69-0.85
Q21	Smoking	96.8	0.84 (0.08)	0.69-0.99
Q22	Past week smoking	99.2	0.80 (0.20)	0.40-1.00

*Q* question, *SE*_*K*_ standard error of kappa, *CI* confidence interval, *NSLBP* non-specific low back pain. Question 2, 6 and 9 are omitted in this analysis

**Table 3:** Test-retest reliability results for questions eliciting numerical responses

		Test	Retest					
Item number	Item description	Mean (*SD*)	Mean (*SD*)	*t*-value	*P* value	ICC	95% CI	SEM
Q2	Mean age of onset of LBP	13.4 (1.96)	13.5 (1.93)	-1.40	0.17	0.87	0.81-0.91	1.36
Q6	Mean intensity for recurrent LBP on VAS	2.59 (1.27)	2.63 (1.30)	-1.00	0.32	0.97	0.94-0.98	0.44
Q9	Mean intensity for ‘point’LBP	2.15 (1.21)	2.69 (1.31)	-1.85	0.09	0.62	0.15-0.86	1.42

*Q* question, *SD* standard deviation, *ICC* Intraclass correlation coefficient, *SEM* standard error of measurement, *CI* confidence interval, *VAS* Visual Analogue Scale, ‘point’=LBP at the point of collecting data

## Discussion

### Content validation

According to experts, the questionnaire showed excellent content validity. This indicates that the questionnaire contain relevant questions that could be used to identify adolescents with recurrent NSLBP and possibly determine the factors associated with the condition. These results should be interpreted with the understanding that the questionnaire was evaluated by four out of the possible seven experts. Although the seven experts would have made the sample representative of the intended professionals in academia and clinical practice, only four timeously responded to the call to participate in the study. The reasons for the lack of participation could not be established but it is largely possible that the experts could have been extremely busy considering the time the content validation study was conducted in December 2015 to March 2016, a period which marks end of the year and the beginning of second final semester at the UZCHS. In anticipation for this, invited experts were given seven days to respond to our request to participate with several reminders being sent through emails. Future studies in our context wanting to include health professionals in academia and clinical practice as content experts may need to ensure that the experts are given sufficient amount of time, probably more than a week, for them to respond to the call to participate and fervently employ other avenues of communication besides emails.

To our knowledge, studies evaluating the content validity of LBP adolescent questionnaires rarely report their procedures and findings. This makes comparisons of findings difficult. Chiwah et al. [22] and Adegoke et al. [54] evaluated the content validity of LBP questionnaires they used in their studies but no information on the procedure and findings were reported. Thus, Staes et al. [45] emphasised the need for such information to be provided for better interpretation of the reported epidemiological findings. It is against that background that results of our study should be interpreted with caution until a replicate study is conducted using a larger sample representative of all the experts concerned with LBP in the country.

### Test-retest reliability

Although certain questions may require some modifications prior to use, test-retest reliability results showed that the present study LBP questionnaire could be used for the investigation of recurrent NSLBP and factors associated among high school-children in Harare, Zimbabwe. Against the background of high prevalence of recurrent NSLBP affecting adolescents in Harare, Zimbabwe, this study may be of epidemiological and clinical importance in that it presents a reliable instrument that could be used in non-clinical settings and in future studies to identify adolescents with LBP and the possible factors associated with the condition. Although there are few published articles in the literature specifically conducted to evaluate the test-retest reliability of developed LBP questionnaires for adolescents, results of the present study are comparable to findings of other studies [28, 30, 45]. Using a similar research design, Bejia et al. [28] consistently reported a wide spectrum of kappa coefficients (k=0.38-1) for their LBP survey questionnaire among 257 Tunisian adolescents aged between 11 and 19 years. Possibly, this consistency is attributable to similarities in the methodology, age of the participants, type of questions asked in the questionnaires and satisfactory item completion achieved between the test-retest studies.

In the present study, high levels of reliability were found for the items that evaluated school bag use, sports participation, lifetime prevalence, smoking status and perceptions of school bag weight. These results suggest that these questions could be appropriate for the investigation of LBP complaints and associated factors in adolescent studies. Comparatively, in a study testing the reproducibility of histories of LBP obtained by self-administered questionnaire from 225 men and women, Walsh and Coggon [55] reported high kappa values for lifetime prevalence (*k*=0.82) and absenteeism from work (*k*=0.76). Although this study was carried in the adult population and the interval period for the test- retest study was 12 months, results on lifetime prevalence confirm that questions eliciting such information are usually reproducible. However, for the present study, the question on school absenteeism which can be equated to the question on work absenteeism from Walsh and Coggon [55] showed moderate reliability. The reason for these differences are not clear but could possibly indicate differences in memory retention between adults and adolescents or differences in the nature and consequences of LBP experienced by adolescents and adults.

Bejia et al. [28] found high levels of reproducibility items that evaluated perceived characteristics of back problems and functional limitations (*k*=0.71-1.00). In comparison, perceived characteristics of recurrent NSLBP for the present study showed similar reliability with questions on frequency and duration of episodes yielding kappa statistic of 0.71 and 0.65, respectively. In addition, the ICC for the pain intensity question was high with a small standard error of measurement. However, in agreement with findings of Walsh and Coggon [55], the screening question for sciatica that could be associated with recurrent non-specific complaints of LBP was less reproducible (*k*=0.20). A pilot study conducted by Chiwaridzo and Naidoo [11] validating a LBP questionnaire among 40 school-children also showed similar results of fair reliability (*k*=0.32). It is possible that concept of sciatica as pain radiating to the lower extremities from the lower back may be difficult to comprehend for adolescents and adults as evidenced by the low reproducibility values. Therefore, it suffices to suggest for careful consideration to be exercised when information related to sciatica is elicited from participants, especially adolescents, in epidemiological studies.

Although the relevance of questions eliciting information on functional consequences secondary to recurrent NSLBP in epidemiological research is undoubtable, the present study found low kappa coefficients for the questions adopted from the Hanover Functional Ability questionnaire enquiring on functional limitations. These results compare with other reports in the literature which reported information on disabilities for everyday activities associated with LBP [55]. The present study elicited functional consequences to recurrent episodes of LBP on activities such as sitting, standing, reaching, bending, walking, running, participating in sports and carrying a school-bag which participating adolescents experienced in the last 12 months. It is possible that the adolescents could have experienced difficulties remembering the exact movements or positions limited by the pain because of the large recall period accounting for the low kappa values.

### Limitations for the study

The results of this study should be interpreted with caution cognisant of a number of limitations. The sample of four content experts who agreed to participate in the validation study was not representative of the intended professionals. In addition, the fact that the experts were tasked to comment only on the relevance of each question as index of validity poses some concerns on the comprehensiveness of the validation of each question. This criterion was recommended by Davies [43] and was adopted for the present study because it was considered simple and quick for the content experts in light of their busy schedules. With all this in mind, there is need for further content validation of the survey questionnaire in our context with increased number of content experts representing all the professions and each evaluating fundamental aspects of sentence construction for the questions such as relevance, clarity, simplicity and ambiguity. In addition, evaluating the content validity of an instrument may not be rigorously sufficient and other forms of validity may need to be addressed such as concurrent or construct validity before the questionnaire is used in large epidemiological studies. For the test-retest study, the common limitations typical of surveys of recall bias and forward telescoping are always a threat to the accuracy of the information reported by participants [56]. It is possible that participants can forget the exact nature and characteristics of the recurrent NSLBP episodes especially when required to recall for a large period of 12 months.

## Conclusion

In terms of the relevance of the questions, the survey questionnaire was found to have excellent content validity by content experts. The questionnaire showed slight to perfect test-retest reliability among high school adolescents. These results calls for further studies assessing the content validity of the questionnaire with increased number of experts representing all the professionals and evaluating several other fundamental parameters of content validation such as clarity, simplicity and ambiguity. In addition, questions which achieved slight reliability such as questions on sciatica and functional limitations should be modified or considered carefully before being used in future studies on adolescent LBP.

## Additional file

Additional file 1: The file can be accessed on the following URL: http://bmcpediatr.biomedcentral.com/articles/10.1186/s12887-015-0518-1. The Adolescent Low Back Pain Questionnaire. (DOCX 185 kb)

## Abbreviations

CI: Confidence Interval
CVI: Content validity index
HRQoL: Health Related Quality of Life
I-CVI: Item Content Validity Index
JREC: Joint Ethics and Research Committee
K: kappa coefficient
LBP: Low Back Pain
MRCZ: Medical Research Council of Zimbabwe
NSLBP: Non-specific Low Back Pain
S-CVI/Ave: Average Scale Content Validity Index
SD: Standard deviation
SMS: Short messaging service
VAS: Visual analogue scale
